# Nighttime Lighting Influences on the Plankton Feeding and Growth of Juvenile Pacific Bluefin Tuna, *Thunnus orientalis*

**DOI:** 10.3390/ani13193128

**Published:** 2023-10-07

**Authors:** Tomoki Honryo, Yoshifumi Sawada, Yasunori Ishibashi

**Affiliations:** 1 Oshima Station, Aquaculture Research Institute of Kindai University, Wakayama 649-3633, Japan; tomoki.honryo@itp.kindai.ac.jp (T.H.); yoshifumi_sawada@kindai.ac.jp (Y.S.); 2 Department of Fisheries, Faculty of Agriculture, Kindai University, Nara 631-8505, Japan; 3 Agricultural Technology and Innovation Research Institute, Kindai University, Nara 631-8505, Japan

**Keywords:** live prey, night-feed, sea cage culture, zooplankton

## Abstract

**Simple Summary:**

This study validated the effect of nighttime lighting on the growth and survival of Pacific bluefin tuna (PBF) juveniles using land-based tanks. As heavy mortality of PBF juveniles occurs in sea cages when they are transferred from nursery tanks, nighttime light can effectively prevent collision deaths. Here, we clearly demonstrate the secondary benefit of nighttime lighting; i.e., nighttime lighting also promotes the growth of PBF juveniles. Various live prey including zooplankton and positive phototoxic larvae aggregate under nighttime lighting at practical sea cage cultures of PBF, allowing the juveniles to take in these feed items even during nighttime. Thus, providing nighttime lighting is an essential technique for increasing the viability as well as growth of PBF juveniles.

**Abstract:**

During fingerling production of Pacific bluefin tuna (PBF) *Thunnus orientalis*, heavy mortality can occur immediately after juveniles are transferred from nursery tanks to sea cages; however, nighttime lighting can moderate this mortality. Additionally, various live prey aggregate due to nighttime lighting in practical sea cage culture of PBF. Here, we investigated whether the growth and viability of PBF juveniles could be improved through promoting feeding on live prey that aggregate under nighttime lighting. Two treatment groups were established using land-based tanks under constant environmental conditions, one in which the juveniles were fed live prey at night (night-feed in four replicate tanks) and the other in which juveniles were not fed during the night (control in four replicate tanks). Although the survival rate did not differ significantly between the two groups, growth was significantly improved in the night-feed group, in which 69–78% of the juveniles showed evidence of feeding during the night. Thus, nighttime lighting plays a vital role in the aggregation of various live prey. PBF juveniles consume these prey in sea cages, which promotes their growth. This may partially serve as a countermeasure against the heavy mortality observed in sea cages.

## 1. Introduction

A project for producing artificially hatched fingerlings of Pacific bluefin tuna (PBF) *Thunnus orientalis* (Temminck & Shclegel, 1844) was launched in the 1970s after several decades of development; these fingerlings successfully completed their life cycle in captivity [1,2]. The development of artificial fingerling production techniques has progressed recently owing to high market demand, decreasing populations in the wild, and regulation of total allowable catch in the past decade [3,4]. Although the survival rate of PBF is not high compared with that of other cultured fish, the grow-out culture of artificial PBF fingerlings has been carried out in a stable manner in recent years [5].

During fingerling production of PBF juveniles, mass mortality has been reported to occur immediately after their transfer from the hatchery to sea cages [6,7]. Okada et al. reported that this mortality is caused by various factors [7]. To prevent these mass mortalities in juveniles, techniques such as adjusting the sea cage diameter [8] and nighttime lighting [9] are used. In addition, previous studies have attempted to elucidate the importance of suitable nighttime lighting duration and light intensity in sea cage culture [10,11].

**Figure 1:**
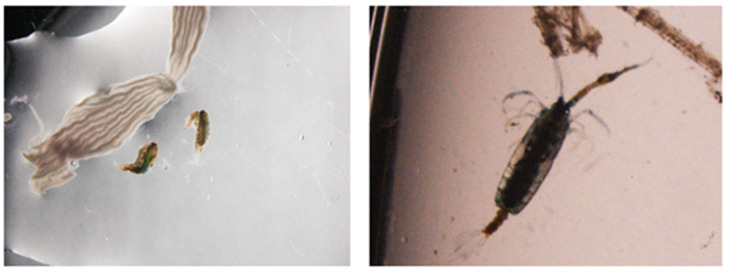
Gut contents of Pacific bluefin tuna (PBF) juveniles captured from sea cages with nighttime lighting (no scales are available). Various zooplanktons were obtained from the stomach contents of PBF juveniles. These photographs were captured by Dr. Masato Kawahara (Aquaculture Technology and Production Center, Kindai University) on 14 August 2016.

Ishibashi et al. reported that nighttime lighting effectively prevents mass mortality in PBF juveniles, owing to their low scotopic vision [9]. Because of their low scotopic vision, PBF juveniles cannot detect the wall of sea cage nets and collide with it, resulting in trauma injuries and death. Thus, nighttime lighting can improve the visibility of nets and serve as an effective countermeasure against collision deaths. Moreover, nighttime lighting is also known to attract various zooplankton, positive-phototaxis larvae, and other biota and improve the survival and growth of seabass *Lates calcarifer* (Bloch) and grouper *Epinephelus tauvina* (Forskal) juvenile in cages [12]. We confirmed that PBF juveniles ingest various zooplankton in sea cages under nighttime lighting (Figure 1). However, the effect of such feed on the growth and viability of PBF juveniles has not yet been investigated yet.

In the present study, we hypothesized that when PBF juveniles are transferred to sea cages, their growth and viability are improved through the intake of prey aggregated due to nighttime lighting in sea cages. Therefore, the aim of this study was to substantiate this hypothesis via conducting a validation experiment.

## 2. Materials and Methods

Environmental conditions in the sea, such as water temperature and tide, are variable. Thus, we anticipated that the type and quantity of aggregated prey under nighttime lighting in sea cages would differ depending on the season and location of the cages. Thus, the reproducibility and reliability of conducting such experiments using sea cages cannot be guaranteed. Hence, in our experiment, we transferred the juvenile PBF to land-based tanks that were not influenced by variable environmental conditions, such as tide and temperature. The experimental fish used in this study were artificially hatched PBF juveniles collected 24 days post hatching [mean total length (TL) = 3.4 cm; mean body weight (BW) = 0.36 g; n = 10] and reared in a 60 m^3^ circular concrete tank. We randomly distributed and stocked 472 PBF juveniles in eight 1 m^3^ tanks (54–65 individuals per tank). During nighttime (18:00–6:00), each experimental tank was provided with 147.8 ± 6.3 lx of nighttime lighting intensity, which was found to be an appropriate intensity by Honryo et al. [13]. An automatic feeder was deployed in each tank during the day (06:00–18:00), through which the fish were fed an appropriate amount (0.91–1.41 mm) of feed (Magokoro Diet Size C comprising 51% crude protein and 14% crude lipid, obtained from Marubeni Nisshin Feed, Tokyo Japan) until they were satiated. Additionally, fish were fed live prey, such as those used in practical fingerling production (e.g., *Artemia*, yolk-sac larvae of *Oplegnathus fasciatus*, and fertilized eggs of *O. fasciatus*), from 09:00–16:00.

The experimental treatment group was given nighttime feed; i.e., fish were fed such live prey at 19:00 and 22:00 (positive treatment; night-feed in four replicate tanks). The control group (negative treatment; control in four replicate tanks) was not fed at night. Treatment without nighttime lighting was not considered in this study owing to heavy mortality under that condition [13]. Growth comparisons are particularly difficult to perform in small tanks without nighttime lighting because 90% of fish die on the third day of rearing [14]. The experimental duration was one week because a high incidence of fish mortality had been reported to occur within one week after transferring fish from nursery tanks to sea cages [7]. The day of transportation was considered as day 1 and the feeding experiment was terminated on day 7. Rearing conditions such as water temperature (°C), dissolved oxygen level (%), and salinity (mg L^−1^) were constant (Table 1). Filtered and UV-treated sea water was provided to each tank at a rate of 2.0 L min^−1^. Dead fish were counted every day when the tank bottom was siphoned for cleaning.

In this study, the growth of the juveniles in the two treatment groups was compared. PBF juveniles had underdeveloped skin and insufficiently formed scales and were very vulnerable to net handling [15]; therefore, a considerable number of PBF juveniles died when their total weight was measured. Therefore, 10 individuals were randomly selected from among the surviving fish in each tank and measured for TL and BW at the end of the experiment. In addition, gut contents were anatomically examined during the night (22:00–23:00) on days 1 (n = 3), 3 (n = 2–4 for night-feed treatment and n = 2–6 for control), and 5 (n = 3). Individual PBF juveniles were captured using a hand net and then immediately euthanized using ice-cold sea water, as described previously [13]. The TL and BW of captured individuals were measured. The abdomen was cut open using scissors and the gut was removed. The stomach and intestine were cut open and their contents were inspected. When the experiment was terminated on day 7, all the surviving fish were captured and counted to calculate the survival rate. The survival rate was calculated using the number of stocked individuals after subtracting the number of sampled fish that were taken to investigate gut contents and the daily dead fish count.

All data are expressed as mean ± standard deviation. Significant differences between the treatments were compared using an independent t-test at *p* < 0.05 using the software SPSS 23 (IBM, Tokyo, Japan).

## 3. Results

**Figure 2:**
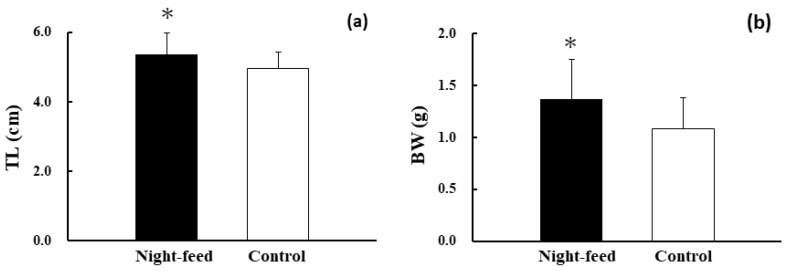
Comparison of total length (**a**) and body weight (**b**) of PBF juveniles at the end of the rearing experiment. Asterisks (*) indicate significant differences between the treatment groups at *p* < 0.05.

The survival rate of the treatment group with night-feeding was found to be 77.6% ± 6.4%, and that of the control group was 76.0% ± 6.2%. However, these values were not significantly different (*p* = 0.755, n = 4). At the end of the experiment, the body sizes of fish from each treatment were calculated: mean TL was 5.35 cm and 4.96 cm for the night-feed treatment and control treatment, respectively, and mean BW was 1.37 g and 1.08 g for the night-feed treatment and control treatment, respectively. TL (*p* = 0.028, n = 40) and BW (*p* = 0.018, n = 40) differed significantly between the two treatment groups. Compared with the control treatment, treatment with night feed resulted in greater TL and BW (Figure 2). Additionally, 69.2–77.8% of the fish in the night-feed treatment group showed clear evidence of ingesting live prey, which contrasted with the fish in the control group, whose stomachs were found to be empty at night (Table 2). It should be noted that almost all individuals had full of prey in their stomach.

## 4. Discussion

Collision death is a typical factor associated with mortality in PBF fingerling production [16]. Low-light-intensity environments, including scotophase, have been reported to induce death in PBF juveniles because of their low scotopic vision [9,17]. PBF juveniles may die from hyperventilation and an imbalance in acid–base regulation when their cruise swimming is disturbed by a collision with the tank wall or net cage [18]. Thus, nighttime lighting plays an important role in preventing collision death through increasing visibility during nighttime, particularly when a suitable light intensity (116 μmol·m^−2^·s^−1^) is used [11]. Therefore, using nighttime lighting has become an essential protocol during the transfer of PBF juveniles from land-based tanks to sea cages [9].

Tuna are typical predators that rely on vision to find their prey, as they lack a well-developed olfactory system [19]. When bright conditions are provided during nighttime in sea cages, PBF juveniles can visually adapt to the light environment [20] and can ingest the feed in sea cages. For instance, the gut contents of PBF juveniles captured from sea cages during night (Figure 1) clearly show that the PBF juveniles consumed the prey that aggregated under nighttime light. Okada et al. reported that insufficient feeding leading to poor growth results in death of PBF in sea cage culture [7]. The results of the present study suggested that feeding during the night did not influence the viability of PBF juveniles; however, growth had significantly improved. In our past visual observation of sea cages located at Kushimoto Bay, Wakayama Prefecture, Japan, the presence of various prey such as zooplankton (mainly copepods) and some positive-phototaxic larvae (species unidentified) was confirmed under nighttime lighting. The larvae of many marine-bottom invertebrates respond positively to light [21]. In addition, it is reported that most zooplankton species undergo diel vertical migration, i.e., the ascent begins near sunset and the descent near sunrise, and there is a descent later in the night [22]. However, it has been reported that the installation of nighttime lighting can disrupt this diel pattern, causing zooplankton that have invaded sea cages to be preyed upon by farmed fish [12]. These gathered zooplanktons, which could be prey items for PBF juveniles, play an important role in promoting growth. The presence of such feed may be subject to constant changes because of tides, seasons, and the location of sea cages. Hence, the exact effect of nighttime live feeding cannot be determined via comparative experiments using sea cages, and we validated our hypotheses using land-based tanks. Nighttime lighting is a useful technique that can directly improve the survival of fish through preventing collision death. Additionally, the present study demonstrated that inducing nighttime feeding via providing live prey improved the growth of PBF juveniles, thereby confirming our original hypothesis. Crucial factors that support this additional benefit of nighttime lighting at sea cages are suitable light intensity (allowing for the congregation of prey), the nutritional values of the aggregated live prey, and the amount of feed ingested by PBF juveniles. Further sustained investigations need to be conducted using sea cages at different locations and seasons in order to indentify the concentration of gathered prey and to fine-tune the techniques associated with PBF juvenile culture.

## Figures and Tables

**Table 1:** Rearing conditions of the two treatment groups.

Treatment	Replications	Temperature (°C)	Dissolved Oxygen (%)	pH	Salinity (mg L^−1^)
Night-feed	4	27.0 ± 0.1	114.8 ± 6.8	8.16 ± 0.04	32.2 ± 0.2
Control	4	27.0 ± 0.1	118.9 ± 5.5	8.20 ± 0.02	32.1 ± 0.3

**Table 2:** The percentage of individuals shown to have ingested live prey during nighttime.

Treatment	Day 1	Day 3	Day 5
Night-feed	77.8%	69.2%	75.0%
Control	0%	0%	0%

## Data Availability

The datasets generated during and/or analyzed during the current study are available from the corresponding author on reasonable request.
